# Maternal Anxiety and Children’s Laboratory Pain: The Mediating Role of Solicitousness

**DOI:** 10.3390/children3020010

**Published:** 2016-06-20

**Authors:** Subhadra Evans, Laura A. Payne, Laura Seidman, Kirsten Lung, Lonnie Zeltzer, Jennie C. I. Tsao

**Affiliations:** 1 School of Psychology, Faculty of Health, Deakin University, 221 Burwood Highway, Burwood Victoria 3125, Australia; 2 Pediatric Pain Program, David Geffen School of Medicine, University of California, Los Angeles, CA 90095, USA; lpayne@mednet.ucla.edu (L.A.P.); lseidman@mednet.ucla.edu (L.S.); kirsten.lung@gmail.com (K.L.); lzeltzer@mednet.ucla.edu (L.Z.); jtsao@mednet.ucla.edu (J.C.I.T.)

**Keywords:** anxiety, children, chronic pain, parenting

## Abstract

There has been limited empirical examination of how parent variables such as anxiety and solicitousness collectively impact child pain response. We sought to examine the relationships among maternal anxiety, solicitous parenting, and children’s laboratory anxiety and pain intensity in children with chronic pain. Participants included 80 children and adolescents (ages 8–18) with chronic pain and their mothers. Children completed questionnaires and lab pain tasks measuring their parents’ solicitous parenting, pressure, cold and heat pain anticipatory anxiety and pain intensity. Using bootstrapping analysis, maternal anxiety predicted child anticipatory anxiety and pain intensity in girls with chronic pain, which was mediated by the child’s report of parental solicitousness. For boys with chronic pain, maternal anxiety predicted boys’ anticipatory anxiety and pain intensity, with no support for mediation. This study adds to the growing literature demonstrating the impact of maternal anxiety on children’s pain. The study highlights the importance of considering parents in treatment designed to reduce children’s pain.

## 1. Introduction

Maternal psychological symptoms and behaviors relate to children’s pain and anxiety. However, research in this area is limited by a lack of clarity in determining the specific parental variables of interest, and in understanding the nature of the relationships between parental behaviors and children’s pain [1]. It is likely that such relationships are complex, involving multiple levels of parental influence, but the specific mechanisms involved in parental transmission of anxiety and pain to children are largely unknown.

Parental influences in children’s pain have been studied in a variety of settings, including children’s dental pain and fear [2], chronic pain [3,4], health-care utilization associated with pain [5] and post-operative pain and distress [6]. The aggregate of these findings points to the coexistence of maternal psychological distress and children’s pain-related distress and pain sensitivity. Even subtle displays of parental fear and anxiety can transmit powerful messages to children regarding the meaning of pain. The mere presence of a parent can cause distress in the child undergoing painful procedures [7]. A mother’s heart rate variability, a marker of stress and autonomic function, two hours preceding their child’s surgery has been found to significantly predict children’s post-anesthetic agitation [8]. In addition, when a parent displays overt symptoms of distress, the impact upon children’s pain and distress may be even more pronounced. For example, children of mothers who were instructed to exaggerate pain during a child observed-cold pressor task had lower pain thresholds compared to no instruction controls [9].

Additional research links specific parenting behaviors to increased children’s pain and anxiety. Solicitous or protective parenting behaviors, which involve positive reinforcement for child pain behaviors such as exempting the child from chores or providing special privileges or attention [10], is one construct that has been studied in the context of child acute and chronic pain. When parents are overly protective, their children show higher distress and report higher bodily pain intensity [11,12]. Children with recurrent abdominal pain are more likely to endorse their parents as using solicitous strategies than are healthy children [13]. One explanation for findings linking parental protective attempts and the subsequent increase in children’s pain is that parental protection may act as a signal to the child that the caregiver is anxious, thus exacerbating the child’s distress [14].

As yet, limited research has mapped out the relationships between maternal anxiety, solicitous behaviors, and children’s pain and pain-related anxiety. Recently, a model found support for the role of parental anxiety and solicitousness in pediatric cancer patients’ pain and quality of life. In this study, parent trait anxiety was a significant predictor of parent solicitous behaviors, as well as a range of parent-reported child pain and quality of life variables in a large sample of children treated for cancer [15]. Although postulated, a meditational model was not examined. An additional study that tested the mediating role of solicitousness in children’s chronic pain reported that parent solicitousness mediated the relationship between parent distress and child functional disability [16]. However, child pain variables were not examined. Thus, few studies reveal how parental variables such as anxiety and solicitousness work together to impact children’s pain or pain-related anxiety.

For parents high in trait anxiety, observing their child’s pain may elicit an aversive state of increased self-oriented distress, leading them to engage in solicitous responses. In a sequential analysis of mothers’ reassurance and children’s postoperative distress, mothers with higher anxiety were more likely to engage in child distress-promoting reassurance behaviors than were mothers lower in anxiety [6]. Relationships between maternal anxiety, solicitousness and child distress and pain may become generalized, and observable in the laboratory. Laboratory paradigms offer the opportunity to test the direct and indirect pathways among these variables in a controlled setting where the intensity and type of pain stimulus can be standardized. An important step is to examine the nature of the relationship between parental distress, solicitousness and child pain outcomes in the laboratory.

**Figure 1:**
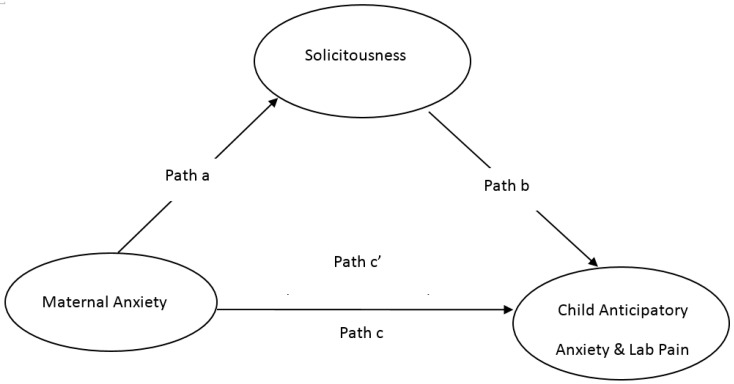
Conceptual model of maternal anxiety, responses to child pain, and dependent child laboratory pain variables.

We sought to test a conceptual model (Figure 1) in children with chronic pain undertaking a series of laboratory pain tasks. It was anticipated that higher maternal anxiety would predict increased maternal solicitous behaviors as perceived by children, in turn predicting increased child laboratory-based anticipatory anxiety and pain intensity in children. We also aimed to test sex differences to understand whether boys and girls with chronic pain responded to maternal influences in dissimilar ways. It was hypothesized that the model would explain the relationship between maternal anxiety and solicitousness for girls, and we wished to explore whether this was the case for boys too. Sex differences seem to exist in the relationship between parent factors and children’s pain, with particular relationships evident between maternal behavior and girls’ pain [17]. In an examination of parental reinforcement of child laboratory-pain, girls whose mothers interacted with them in a pain-promoting manner reported more cold-pressor pain than girls of mothers who received no training. Girls of mothers who received pain-reducing training reported the least amount of pain. No relationships were evident for boys. [18]. An additional study showed that the effect of maternal attention was greater on daughters’ gastrointestinal symptoms than on sons’ symptoms [19].

## 2. Methods

### 2.1. Participants

The sample for this study was part of a larger study examining sex and pubertal relationships to pain in healthy children and those with chronic pain. Participants in the present study included 80 children and adolescents (ages 8–18) with chronic pain and their mothers, recruited through a multidisciplinary, tertiary clinic specializing in pediatric chronic pain (approximately 10% of the sample were recruited though craigslist postings). Inclusion criteria followed the commonly accepted definition of pain persisting for three months or longer [20]. Each diagnosis of a chronic pain condition was confirmed by a pediatrician specializing in chronic pain (Dr. Lonnie Zeltzer.).

Eligibility was confirmed by telephone. Parents were asked whether their child met any of the following exclusionary criteria: acute illness/injury that may impact laboratory performance (e.g., fever) or that affects sensitivity of the extremities (e.g., Raynaud’s disease); daily use of opioids; recent traumatic experiences; developmental delay; autism; or significant anatomic impairment that could preclude participation in pain induction. Three hundred and sixty-four families were screened for eligibility by telephone: eight families (five control, three pain) were excluded as a result of acute injury/illness, recent traumatic experiences, or developmental delay/autism. Of the 138 families of children with pain who called to participate, 58 declined to participate due to lack of interest or scheduling difficulties.

The average age of children in the final sample was 13 years and 8 months (52 girls and 28 boys), and the average age of mothers was 45 years. The majority of mothers possessed some college qualifications, and the majority of children were non-Hispanic white (see Table 1).

Written informed consent forms were completed by parents, while children and adolescents completed written assent. The study was approved by the University of California, Los Angeles (UCLA, Los Angeles, CA, USA) institutional review board. Each family member received $50 cash for participating.

### 2.2. Procedures

The study protocol and laboratory pain tasks have been described in previous reports relating to the larger study [21,22,23]. Briefly, children and their mothers were escorted to separate rooms to complete questionnaires using an online survey system; there was no contact between children and their mothers during the entire laboratory session. Children were then escorted to the laboratory where they were instructed on the use of the 0–10 Numerical Rating Scale (NRS, described in the measures section), and completed four laboratory pain tasks: an evoked pressure task, a cold pressor tolerance task, a focal pressure tolerance task, and a conditioned pain modulation (CPM) task (see task descriptions below). Tasks were completed in the same order since previous research showed no order effects [24]. The laboratory session took 30–45 min.

#### 2.2.1. Evoked Pressure (EP) Task

To assess pressure pain sensitivity, we used the Gracely procedure [25], in which discrete 5 s pressure stimuli were applied to the fixated thumbnail of the left hand with a 1 × 1 cm hard rubber probe. The rubber probe was attached to a hydraulic piston, which was controlled by a computer-activated pump to provide repeatable pressure-pain stimuli of rectangular waveform. First, a series of stimuli was presented in a predictable, ascending manner, beginning at 0.066 kg/cm^2^ and increasing in 0.132 kg/cm^2^ intervals up to the participant’s report of moderate pain (a 6 Numeric Ratings Scale (NRS) rating) or to a maximum of 1.12 kg/cm^2^ (evoked pressure–ascending, EP ASC). Next, stimuli were delivered at 15 s intervals in random order using the multiple random staircase (evoked pressure–multiple random staircase, EP MRS) pressure-pain sensitivity method [26].

#### 2.2.2. Cold Pressor Tolerance (CPT) Task

A single trial of the cold pressor was administered during which participants placed the right hand in a cold pressor unit comprising a Techne TE-10D Thermoregulator, B-8 Bath, and RU-200 Dip Cooler (Techne, Burlington, NJ, USA). The unit maintained the water at a temperature of 5 °C and kept the water circulating to prevent localized warming around the hand. Recent research has demonstrated 5 °C to be an appropriate temperature for use with children and adolescents in the age range of the current study [27], and a recent systematic review suggests that using a temperature of less than 10 °C in children older than eight years may reduce the likelihood of a ceiling effect [28]. Participants’ hands were submerged up to approximately 2 inches above the wrist. Participants were instructed to keep their hand in the cold water for as long as they possibly could but that they could terminate the trial at any time. The task had an uninformed ceiling of 3 min.

#### 2.2.3. Tonic Pressure Tolerance (TPT) Task

The Ugo Basile Analgesy-Meter 37215 (Ugo Basile Biological Research Apparatus, Comerio, Italy) was used to administer a single trial of focal pressure through a dull Lucite point approximately 1.5 mm in diameter to the second dorsal phalanx of the middle finger of the right hand. Participants were instructed to keep their finger under the pressure for as long as they possibly could, but that they could terminate the trial at any time. The task had an uninformed ceiling of 3 min.

#### 2.2.4. Conditioned Pain Modulation (CPM) Task

The conditioned pain modulation task involved a test stimulus (TS) of phasic pressure stimuli delivered to the left thumbnail, and a conditioning stimulus (CS) of immersion of the right hand in 5 °C water [23]. The amount of pressure used as the TS was determined by averaging the final four pressure steps of the EP MRS high staircase. This value represents the amount of pressure that would reliably induce a rating of moderate pain in that participant. Participants were instructed to leave their hand in the water for 30 s and told that they would be informed by the research assistant when this time period had elapsed; participants were also told that they could remove their hand before the end of the 30 s if it became too uncomfortable/painful.

### 2.3. Measures

#### 2.3.1. Pain Task Measures

*Anticipatory anxiety:* was assessed with a 0 (none) to 10 (worst or most possible) NRS after task instructions had been given, and before each task had commenced (*i.e.*, “now that you know what you will be doing, how nervous, afraid, or worried are you about the task”).

*Pain Intensity:* was assessed using the same 0–10 NRS after each task. Participants were instructed that 0 meant “none” and 10 meant the “worst or most pain possible”. The 11-point NRS has been demonstrated to be a reliable and valid method of assessing self-reported pain intensity in children as young as 8 years old [29].

#### 2.3.2. Self-Reporting Questionnaires

The Anxiety subscale of the Brief Symptom Inventory-18-Item Version (BSI-18) [30] were used to assess mothers’ anxiety. Mothers rated their own symptoms, with six items rated on a 5-point scale from “not at all” to “extremely”. Responses are summed to obtain the total anxiety subscale score. The BSI-18 has demonstrated reliability and validity [30]. The cronbach’s alpha for the present sample was 0.78.

Children’s perceptions of their mother’s solicitousness was assessed by the Protect subscale of the Adults’ Responses to Children’s Symptoms (ARCS) Child Report version [31,32]. The subscale is comprised of 15 items assessing the extent to which children perceive that their parents respond to their child’s pain by placing the child in the “sick role” (e.g., limiting child’s activities, granting special privileges, *etc.*) [31]. Responses are scored from 0 (never) to 4 (always); subscale scores are calculated by averaging the individual item responses. Higher scores indicate more of that behavior. Instructions were modified to inquire about parent behaviors when the child experienced pain as opposed to the original instructions which were focused on stomachaches. The child report version of the ARCS has a demonstrated alpha reliability of 0.86 and has shown to be correlated to the analogous parent report measure [32]. The cronbach’s alpha for the present sample was 0.83.

### 2.4. Data Analysis

Analyses were conducted using SPSS 22. Data were screened to identify outliers and verify normality distribution. Descriptive analyses were conducted, including presentation of the sample, and differences between boys and girls were examined, using Chi squares, and *t*-tests where appropriate. Correlations were then performed between maternal and child variables separately for boys *versus* girls, followed by regression analyses.

Regression analyses were conducted to: (1) examine the relation between maternal anxiety and child anticipatory anxiety/pain intensity; (2) the relation between maternal anxiety and solicitousness; and (3) the relation between solicitousness and child anticipatory anxiety/pain intensity. Upon establishment of these conditions, bootstrapping analyses were then used to test the mediation hypotheses [33]. All analyses controlled for age. Bootstrapping involves repeatedly randomly sampling observations with replacement from the data set to compute the desired statistic in each resample. Indirect effects were analyzed based on 5000 bootstraps and were evaluated as significant if the 95% bias-corrected confidence interval of the indirect effect does not include zero. Consistent with recent views on mediation [34,35,36], we consider mediation analyses even in the absence of a direct effect. For example, Rucker and Preacher [34], argue that significant indirect effects can occur in the absence of significant total or direct effects.

## 3. Results

### 3.1. Descriptive Statistics

Demographic data, including sex, age, and race/ethnicity, are presented in Table 1. Presenting pain diagnoses were: 61.3% headaches (migraines and myofascial, vascular, tension, stress-related, or other type of headaches); 46.3% functional neurovisceral pain disorder (functional bowel, uterine, or bladder disorder); 33.8% myofascial pain (of any part of the body excluding headaches); 26.3% fibromyalgia; 10.0% complex regional pain syndrome; and 10.0% joint pain (note that percentages sum to more than 100% due to multiple pain diagnoses). Multiple pain diagnoses were present in 61.3% (*n* = 49) of the sample. Frequencies of each pain diagnosis by sex are presented in Table 1. Chi-squared analyses revealed no significant differences in diagnosis rate between sexes. The only significant group difference to emerge was for child age; girls with chronic pain were significantly older than boys.

Means and standard deviations for each of the maternal variables and child lab pain variables are displayed in Table 2. Maternal anxiety means were within population norms. There were no significant sex differences on the maternal or child questionnaire or laboratory variables.

As presented in Table 3, correlations between the maternal and child variables were performed for boys and girls on each of the laboratory pain task variables. For girls, maternal anxiety was significantly related to child anticipatory anxiety (for EP *r* = 0.45, *p* = 0.00; for CPT *r* = 0.45, *p* = 0.00; for TPT *r* = 0.42, *p* = 0.01; for conditioned pain modulation (CPM) *r* = 0.45, *p* = 0.00), while solicitousness was significantly associated with most child lab pain variables (for CPT *r* = 0.35, *p* = 0.01; for TPT *r* = 0.27, *p* = 0.049; for DNIC *r* = 0.32, *p* = 0.03). For boys, maternal anxiety was associated with child lab anxiety (for EP *r* = 0.38 *p* = 0.047; for DNIC *r* = 0.61, *p* = 0.00) and child lab pain (EP *r* = 0.41, *p* = 0.03; for CPT *r* = 0.57, *p* = 0.00; for TPT *r* = 0.53, *p* = 0.000; for DNIC *r* = 0.44, *p* = 0.03), whereas maternal solicitousness was not associated with any child variables. Age was not correlated with any of the maternal or child variables for girls with pain; however, since girls with pain were significantly older than the other sub-groups, age was included as a covariate in hypothesis testing. For boys with pain, age was significantly related to both maternal anxiety (*r* = −0.40, *p* < 0.05), and solicitousness (*r* = −0.43, *p* < 0.05), suggesting that, as they get older, boys perceive their mothers as using fewer solicitous responses, and mothers rate themselves as less anxious. Age was thus included as a covariate in all hypothesis testing.

### 3.2. Hypothesis Testing

Average summary measures across all four laboratory tasks were created to produce a single score for each anticipatory anxiety and pain intensity. Hypothesis testing was performed on these summary measures controlling for child age.

Results for the regression analyses for girls with chronic pain are presented in Table 4. First, there was support for the relationship between maternal anxiety and girls’ anticipatory anxiety (*β* = 0.55, *p* = 0.00). However, no significant direct effect of maternal anxiety on girls’ lab pain intensity; Second, there was support for the relation between maternal anxiety and solicitousness, such that greater maternal anxiety was associated with greater use of mothers’ solicitous responses for girls with chronic pain (*β* = 0.28, *p* = 0.048); Third, there was support for the relation between solicitous parenting and girls’ anticipatory anxiety and pain intensity, such that greater use of solicitous responses was associated with greater anticipatory anxiety (*β* = 0.39, *p* = 0.01) and pain intensity in girls (*β* = 0.36, *p* = 0.01).

For boys (as presented in Table 4), there was support for the relationship between maternal anxiety and boys’ outcomes. Greater maternal anxiety was a significant predictor of boys’ anticipatory anxiety (*β* = 0.60, *p* = 0.00) and pain intensity (*β* = 0.44, *p* = 0.00), explaining 36% and 19% of the variance respectively. However, there was no support for the relation between maternal anxiety and solicitousness. There was also no support for the relation between solicitous parenting and boys’ pain outcomes. Thus, further mediation analyses for boys were not undertaken.

**Figure 2:**
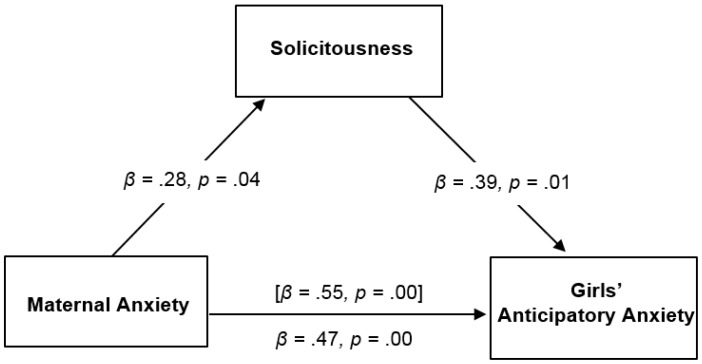
Mediating role of solicitousness in the relation of maternal anxiety with lab anticipatory anxiety for girls with chronic pain. Estimates of total effect of maternal anxiety on girls’ pain outcomes are presented in brackets, with values representing estimates of the total indirect effect of maternal anxiety on girls’ pain outcomes through maternal solicitousness presented below.

**Figure 3:**
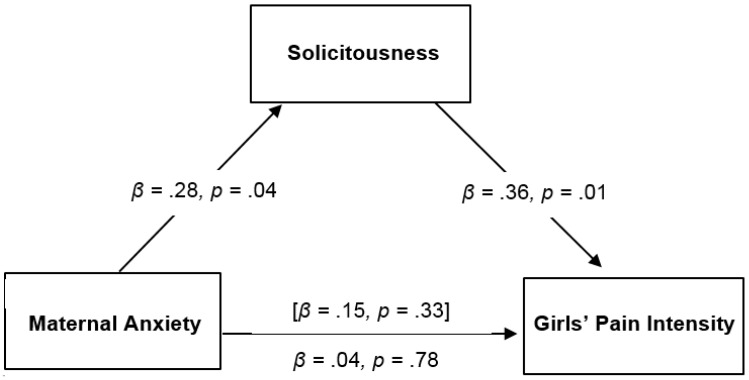
Mediating role of solicitousness in the relation of maternal anxiety with girls’ lab pain intensity. Estimates of total effect of maternal anxiety on girls’ pain outcomes are presented in brackets, with values representing estimates of the total indirect effect of maternal anxiety on girls’ pain outcomes through maternal solicitousness presented below.

Bootstrapping analyses were conducted with solicitousness as a hypothesized mediator between maternal anxiety and anticipatory anxiety and pain intensity in girls only. Mothers’ solicitousness mediated the relation between maternal anxiety and girls’ anticipatory anxiety (see Figure 2). The direct effect of maternal anxiety on girls’ anticipatory anxiety was reduced when solicitousness was included as a mediator. This result was supported by bootstrapping analyses, which showed the bootstrapped indirect effect was 0.05 (95% bootstrap confidence interval (BCI) = 0.01, 0.14) for girls’ anticipatory anxiety. For girls’ pain intensity, there was evidence of an indirect effect. When maternal anxiety and solicitousness were entered in the model together, they significantly predicted girls’ pain intensity, yet maternal anxiety alone did not predict girls’ pain intensity. Consistent with recent writings on mediation [34], significant indirect effects can occur in the absence of significant direct effects, often due to a suppression effect of a third variable, or a small sample size. The significance of the indirect effect of solicitousness on the relationship between maternal anxiety and girls’ lab pain intensity was supported by bootstrapping analyses, which showed the bootstrapped indirect effect was 0.06 (95% BCI = 0.02, 0.13). Figure 2 and Figure 3 summarize the significant mediating results.

## 4. Discussion

This study sought to investigate the relationship between maternal anxiety and children’s laboratory anticipatory anxiety and pain intensity in boys versus girls with chronic pain, and to test the hypothesis that these relationships were mediated by children’s reports of maternal solicitous behavior. There were no sex differences in the degree to which children perceived their mothers as solicitous across the groups; mothers reported similar levels of anxiety; and maternal anxiety emerged as a significant predictor of both girls’ and boys’ laboratory pain and anxiety. However, there were differences in the relationships among these variables between the sexes. In particular, perceptions of mother’s solicitous behavior mediated the relationship between maternal anxiety and child lab pain for girls only.

Our findings are consistent with the existing literature demonstrating links between parental distress and behaviors, and children’s pain sensitivity and anxiety. For example, Logan and Scharff (2005) [37] found that maternal psychological distress was predictive of functional disability in children with chronic pain; moreover, mothers in the study were similar to the mothers in the present study in that they were not significantly more distressed than healthy populations. Other lab studies have reported that children of mothers that were instructed to exaggerate pain during a child observed-cold pressor task had lower pain thresholds compared to controls whose mothers were given no specific instructions [9]. The present study suggests that the relationship between maternal distress and child pain and pain-related anxiety may occur through maternal solicitousness for girls with chronic pain.

Our findings go beyond what is currently known about parental influences in children’s pain and pain-related anxiety. Few studies have examined how parental variables interact in influencing children’s pain. For example, in a recent study, parent trait anxiety emerged as a significant predictor of parent solicitous behaviors, parent-reported child pain, and child quality of life variables in children treated for cancer [15]; however, a model describing the nature of the relationships between the variables was not offered. In one mediation study, it was reported that parent protectiveness mediated the relationship between pain-specific parent distress and child functional disability [16]. The present study extends these findings to the laboratory, in which it is possible to measure the immediate effects of pain stimuli, including behavioral and emotional responding.

Overall, parental solicitousness has emerged as an important variable in understanding and treating the etiology and maintenance of chronic pain in children. For example, studies have shown that a brief cognitive behavioral therapy intervention, involving reducing parental solicitousness as one its components, resulted in pain reduction and decreased disability for children with chronic pain in both short- and long-term follow-up [4]. The present study is the first time that a meditational model for solicitousness has been demonstrated for the relationship between parental anxiety and child laboratory-based pain and anxiety.

This is also the first study where a meditation model has been examined in a sex specific manner. Consistent with our previous work, we report a greater influence of maternal symptoms on girls’ pain outcomes than on boys’ outcomes. In earlier studies, we demonstrated relationships between maternal symptoms (anxiety sensitivity, fear of pain, anxiety and bodily pain) and healthy girls’ laboratory pain responses, but fewer relationships with boys’ pain responses (Tsao, Lu, Myers *et al.*, 2006; Evans *et al.*, 2008; Evans *et al.*, 2010). For example, parent anxiety sensitivity was related to child laboratory pain intensity through its contribution to anxiety sensitivity in girls, but not in boys (Tsao, Lu *et al.*, 2006). Together, these findings highlight the particular influence of mothers on daughters’ pain and the importance of examining sex differences in the context of pediatric pain.

A number of study limitations are worth noting. Our cross-sectional findings prohibit an understanding of the direction of the relationships. Does child pain elicit a solicitous parental reaction, or do certain parental styles elicit pain in children? Perhaps higher levels of negative affect in children with chronic pain drive the relationship. Other potential mediators, such as parental catastrophizing should be examined, especially given findings that catastrophizing may mediate the relationship between solicitous parenting and children’s pain behaviors [38]. Future longitudinal analyses may reveal causal associations among a number of such constructs. In addition, since this study only included mothers, the impact of fathers both directly on daughters’ and sons’ anxiety and pain responses and indirectly through impact on the mother/child pain relationship was not explored. The lack of findings of mothers’ solicitousness as a mediator of the relationship between maternal anxiety and child anxiety/pain relationships for sons, compared to that found for daughters, suggest that there may be other factors that mediate the mother/son pain relationship not explored in this study. It may also be the case that, since there were fewer boys in the study, the lack of mediation was due to lower power in the sample of boys. Possible moderators to examine in the future include duration and severity of the child’s condition and whether the parent has chronic pain. While a strength of the sample is the blending of disease groups to allow for examination of psychological and family processes in a broad group of children chronic pain, it will be important in future research to explore the impact of specific disease-related factors such as duration of diagnosis on child and family outcomes. The present findings contribute to the literature on a broad population of children with chronic pain and reveal complex models of pain, maternal anxiety, parenting and laboratory based pain in families dealing with children who have chronic pain.

## 5. Conclusions

Children with chronic pain may benefit from a multifaceted intervention, including pain management strategies, treatment targeted to depressive or anxiety symptoms to improve coping skills, and parents and other family members may be taught adaptive, non-reinforcing responses to both distress and pain behaviors in their children to optimize child functioning (Sieberg *et al.*, 2011). Our findings highlight the importance of considering parental factors, and especially maternal anxiety when evaluating and treating children with chronic pain. Maternal anxiety appears to impact boys and girls through different mechanisms. Maternal solicitousness may be particularly important when understanding girls’ anxiety and pain intensity, whereas maternal anxiety may affect boys’ anxiety and pain through other, as yet untested, influences. It is possible that girls with chronic pain are highly sensitive to modeling of anxiety and reinforcing behaviors in their mothers, given findings that girls with pain tend to become heavily enmeshed and reliant on their mothers, whereas boys with pain may respond to maternal worries with increased efforts for independence [39]. Future studies should continue to focus on the impact of maternal emotional and behavioral contributors to boys’ and girls’ pain, as well as explore the role of fathers’ emotional and behavioral functioning in boys and girls with chronic pain. Our findings relating to sex differences, if verified by further research, suggest that clinical efforts for girls with chronic pain should invest in mothers, and include strategies for reducing solicitous parenting that reinforces child pain behaviors.

## Figures and Tables

**Table 1:** Demographic information.

	Pain Boys (*n* = 28)	Pain Girls (*n* = 52)
**Child Age (mean (SD))**	12.72 (2.35)	14.92 * (2.45)
**Pain Group Diagnoses (*n* (%))**		
Headaches	16 (57.1)	33 (63.5)
Neurovisceral	12 (42.9)	25 (48.1)
Myofascial (non headache)	10 (35.7)	17 (32.7)
Fibromyalgia	4 (14.3)	17 (32.7)
CRPS	3 (10.7)	5 (9.6)
Joint pain	3 (10.7)	5 (9.6)
**Child Ethnicity (*n* (% of subgroup))**		
Non-Hispanic/Latino	21 (75.0)	36 (69.2)
Hispanic/Latino	7 (25.0)	16 (30.8)
**Child Race (*n* (% of subgroup))**		
White	17 (60.7)	37 (71.2)
African-American	6 (21.4)	5 (9.6)
Asian	1 (3.6)	1 (1.9)
American Indian/Alaska Native	0 (0.0)	0 (0.0)
Multi-Racial	4 (14.3)	9 (17.3)
Mother age [mean (SD)]	44.70 (5.29)	45.62 (6.63)
**Mother Education Level (*n* (% of subgroup))**		
High school graduate or below	4 (14.3)	8 (15.4)
Some college/AA degree	8 (28.6)	12 (23.1)
College graduate (BA/BS)	8 (28.6)	15 (28.8)
Post-graduate degree	8 (28.6)	17 (32.7)

Note: * indicates significant difference from other groups, *p* < 0.05.

**Table 2:** Means (standard deviation, SD) of maternal psychosocial variables and child laboratory pain variables for boys and girls.

	Pain Boys	Pain Girls
Mother BSI Anxiety Subscale	2.68 (3.14)	3.04 (3.11)
Child ARCS Protect Subscale	2.02 (0.71)	1.63 (0.58)
EP Ant Anx	3.07 (2.36)	2.92 (2.65)
EP Pain	6.04 (2.52)	6.51 (1.38)
CPT Ant Anx	1.61 (1.57)	1.98 (2.29)
CPT Pain	5.89 (2.56)	5.75 (2.90)
Pressure Ant Anx	4.07 (2.73)	3.18 (2.46)
Pressure Pain	5.78 (2.78)	6.02 (2.42)
CPM Ant Anx	3.68 (2.77)	3.21 (2.64)
CPM Pain	6.13 (2.85)	6.09 (2.90)

Note: BSI = Brief Symptom Inventory-18-Item Version; ARCS = Adults’ Responses to Children’s Symptoms; EP = Evoked Pressure task; CPT = Cold Pressor Tolerance task; CPM = Conditioned Pain Modulation task; Ant Anx = Anticipatory Anxiety.

**Table 3:** Correlations among maternal and child lab variables.

	Maternal Anxiety	Solicitousness
**Girls with Pain**		
Maternal Solicitousness	0.28 *	
***Child Lab Pain***		
EP- Anticipatory Anxiety	0.45 **	0.31 *
EP- Pain Intensity	0.02	0.20
CP- Anticipatory Anxiety	0.45 **	0.34 *
CP- Pain Intensity	0.12	0.35 *
TPT- Anticipatory Anxiety	0.35 *	0.30 *
TPT- Pain Intensity	0.01	0.27 *
CPM- Anticipatory Anxiety	0.45 **	0.40 *
CPM- Pain Intensity	0.20	0.33 *
**Boys with Pain**		
Maternal Solicitousness	−0.03	
***Child Lab Pain***		
EP- Anticipatory Anxiety	0.38 *	0.09
EP- Pain Intensity	0.41 *	−0.30
CP- Anticipatory Anxiety	0.17	0.03
CP- Pain Intensity	0.57 **	−0.12
TPT- Anticipatory Anxiety	0.27	0.29
TPT- Pain Intensity	0.53 **	0.23
CPM- Anticipatory Anxiety	0.61 **	−0.15
CPM- Pain Intensity	0.44 *	−0.11

EP: Evoked Pressure; CP: Cold Pressor; TPT: Tonic Pressure Tolerance; CPM: Conditioned Pain Modulation; * *p* < 0.05, ** *p* < 0.01

**Table 4:** Regression models for pre-conditions of mediation.

Maternal Predictors & Child Pain	Child Anticipatory Anxiety			Child Pain Intensity			Solicitousness		
	*β*	*t*	*R² Δ*	*β*	*t*	*R² Δ*	*β*	*t*	*R² Δ*
**Girls with Chronic Pain**									
Child Age	−0.26	−10.79	0.07	0.02	0.14	0.00	0.04	0.301	0.001
Maternal Anxiety	0.55	4.60 **	0.30	0.15	10.0	0.02	0.28	2.03*	0.08
Solicitousness	0.39	2.95 **	0.15	0.36	2.6 *	0.13			
**Boys with Chronic Pain**									
Child Age	−0.27	−1.4	0.08	−0.17	−0.81	0.03	−0.43	−2.41 **	0.18
Maternal Anxiety	0.60	3.68 **	0.36	0.44	2.21 *	0.19	−0.23	−1.21	0.05
Solicitousness	0.03	0.12	0.01	−0.19	−0.73	0.03			

All models controlled for child age, * *p* < 0.05; ** *p* < 0.01, *β* denotes standardized beta coefficients.
